# Integrated Electrowetting Nanoinjector for Single Cell Transfection

**DOI:** 10.1038/srep29051

**Published:** 2016-07-04

**Authors:** Elaheh Shekaramiz, Ganeshkumar Varadarajalu, Philip J. Day, H. Kumar Wickramasinghe

**Affiliations:** 1 Department of Biomedical Engineering, University of California Irvine, California, USA; 2 Department of Electrical Engineering, University of California Irvine, California, USA; 3 Manchester Institute of Biotechnology, the University of Manchester, Manchester, UK

## Abstract

Single cell transfection techniques are essential to understand the heterogeneity between cells. We have developed an integrated electrowetting nanoinjector (INENI) to transfect single cells. The high transfection efficiency, controlled dosage delivery and ease of INENI fabrication promote the widespread application of the INENI in cell transfection assays.

The need for techniques to deliver DNA and other macromolecules into cells has been steadily increasing since the advent of gene therapy applications^1^. Many techniques have been introduced and evolved in recent decades for this purpose both at the bulk cell level as well as single cell level^2^. The most common transfection techniques at the bulk cell level are viral and chemical transfections^3^. Although viral transfection has high transfection efficiency, it is very expensive and can cause inflammatory responses^1^,^4^,^5^. Chemical transfection techniques on the other hand have low transfection efficiency^6^. Both viral and chemical techniques lack control of dosage and can not deliver genes to cells individually^2^. Single cell transfection techniques have limited transfection efficiency, dosage control, cell viability, and ease of fabrication^7^. For instance, microinjection, which is the most common transfection technique, damages cells due to the large microinjector needle diameters of 0.5–5 μm^8^ and the needles tend to clog before injection^9^. Other single cell transfection techniques such as opto-poration lack controlled dosage delivery and need optimization of parameters for each cell type^10^,^11^. Single cell electroporation techniques have low cell viability given the high electric fields applied to each cell^12^. Grandbois *et al*. transfected cells by inserting plasmid-decorated Atomic Force Microscopy (AFM) probes and incubating them in cells for one minute. The number of molecules injected was not quantified and the efficiency was 30%^2^. Recently, Vorholt *et al*. injected plasmids into single cells using specialized AFM tips fabricated with integrated fluidics^13^. However, the fabrication of such AFM tips is cumbersome and they possess a reported efficiency of 40%^13^. Most importantly, injection volumes could only be determined after injection with the help of a co-injected fluorescent marker^13^. Pourmand’s group used double-barrel nanopipettes to inject fluorescent dyes inside human BJ fibroblast cells^2^. Mirkin *et al*. also developed a method where one electrode was placed inside culture media and another inside the micropipette and used it to deliver positively and negatively charged fluorescent dyes into cells^14^. However, neither techniques were used to change the genetic expression of living cells^7^,^14^.

In the current study, we present a new simple transfection method using an integrated electrowetting nanoinjector (INENI) with controlled dosage delivery and high transfection efficiency. INENIs were fabricated by coating a layer of iridium/platinum (Ir/Pt) on the outside of a drawn capillary and inserting an AgCl electrode inside the drawn pipette containing 1,2-dichloroethane (DCE). The INENIs were calibrated for the femtoliter volume of solution with respect to applied voltages. This calibration data is compared to proposed theory of the technique. Following calibration, the INENIs were used to deliver femtoliter volumes of macromolecules such as plasmids inside single cells. Lastly, the transfection efficiency of plasmid injection was verified.

## Results

**Figure 1:**
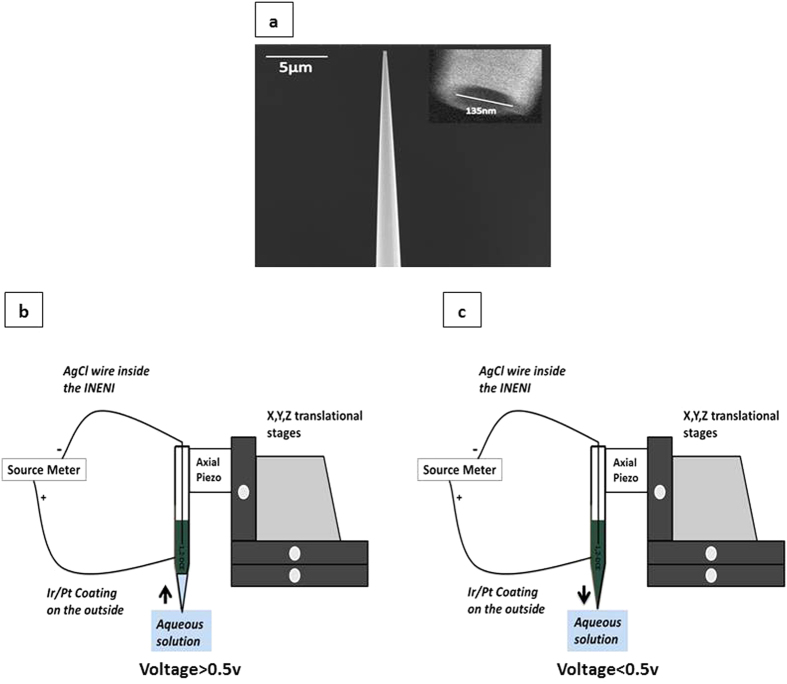
(**a**) Upper Left Panel. Scanning Electron Microscopy (SEM) image of INENI. (**b**) Lower Left Panel. Schematics of INENI device for pick up, where voltages higher than 0.5v produces solution uptake. (**c**) Lower Right Panel. Schematics of INENI device for ejection., where voltages lower than 0.5v gives rise to solution ejection.

The capillaries were drawn and coated with a 30 nm layer of Ir/Pt. The INENI openings were roughly 139 nm ± 3.5 nm compared to commercial microinjection tips (0.5–5 μm) (Fig. 1).

**Figure 2:**
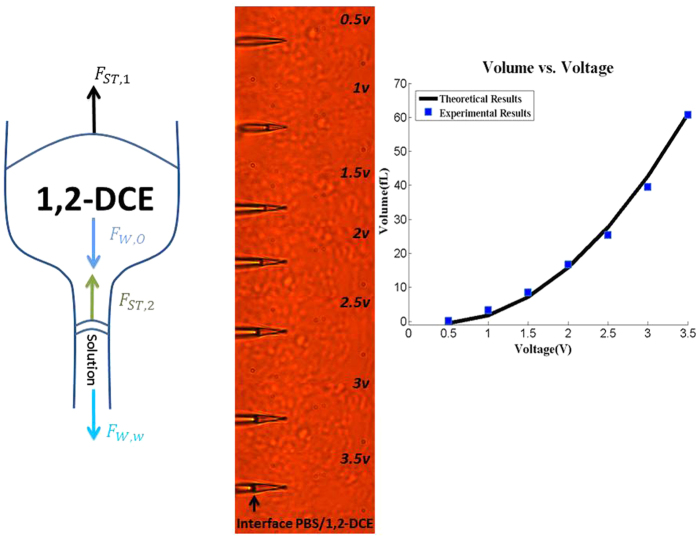
Study of Injected Volume versus Applied Voltage. Left Panel. Schematic of governing forces in an INENI. Middle Panel. Image of PBS/1,2-DCE meniscus for different applied voltages. Right Panel. Comparison of theoretically predicted ejected volume with experimental data.

With positive bias applied on the outer Ir/Pt coating layer and a silver wire electrode inside 1,2-DCE, small volumes can be injected or picked-up from a single cell depending on magnitude of voltages applied. To quantify experimentally the fluid volume uptake with respect to applied potential difference, different voltages were applied and the corresponding increase in fluid height within the INENIs were measured using a known distance as a reference with ImageJ software. The radius corresponding to the height of liquid was calculated based on the angle of the cone. The cone angle from SEM images was measured to be 4.3 degrees and average radius was 70.2 nm. Higher applied voltage was related to increased fluid uptake as noted in Fig. 2 (blue markers).

The proposed theory of this technique is as follows. An equation to balance the forces acting on the liquid column inside INENIs is given below:


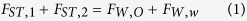


where,

*F*_*ST*,1_ = 2*πrγ*_*L,V*_Cosφ, force due to surface tension between liquid- vapor in a capillary^15^

*F*_*ST*,2_ = 2π*rγ*_*L,L*_ Cosθ, force due to surface tension between liquid- liquid in a capillary^15^

*F*_*W*,*O*_ = *ρ*_*O*_g*v*_*O*_, weight of organic phase

*F*_*W*,*w*_ = *ρ*_*w*_g*v*_*w*_, weight of solution

Based on Lippmann’s equation^16^,


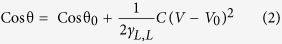


And,

*γ*_*L,V*_ is the surface tension between 1,2-DCE/vapor (38.7 mN/m),

*γ*_*L*,*L*_ is the Surface tension between water/1,2-DCE (28.2 mN/m)^17^,

φ _0_ is the wetting angle between organic phase and glass (135.9 °C)^18^,

θ is the variable angle with applied voltage between water and glass,

θ_0_ is the angle between water and glass without applied voltage (10 °C),

*v*_o_ is the volume of organic phase (49fL),

*v*_*w*_ is volume of water,

*ρ*_*O*_ is density of organic phase (1.25(*g*)/(*cm*^3^))^19^,

*ρ*_*w*_ is density of water (1(*g*)/(*cm*^3^)),

r is the radius of INENI cylinder (150 nm),

*V*_0_ is potential at the point of zero charge (0.37 v)^17^,

V is the applied voltage, C is the capacitor of double layer per unit area^20^. Assuming constant capacitor, the capacitance of double layer at the point of zero charge is 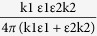, where k_1_^−1^ (0.7 nm)^21^ and k_2_^−1^ (calculated to be 30 nm) are the Debye length of the two immiscible solutions, and ε_1_ and ε_2_ are the permittivities of water (7.1 × 10^−10^) and 1,2-DCE (8.9 × 10^−11^) respectively^21^,^22^.

Combining equation 1 and 2, we get,


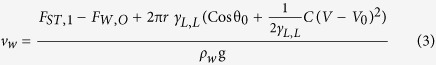


with sufficiently high voltage, a force is generated to move the solution inside an INENI. There is a force due to surface tension acting on the organic phase which is constant. This force is dependent on the wetting angle of organic phase with glass, and the surface tension between vapor/organic phase. The weight of the organic phase and the aqueous solution are the two other forces (Fig. 2) that must be considered for the INENI force balance equation. While the weight of organic phase is constant, the weight of aqueous solution will depend on how much liquid remains inside the INENI. Lastly, there is a force due to surface tension acting on the aqueous solution. A double layer forms between the two immiscible solutions of 1,2-DCE and electrolyte. In this study, the capacitor for the double layer was considered constant and the capacitor at the point of zero charge was used in the equation^22^.

The theory (solid line in Fig. 2) matches closely with the experimental data (markers in Fig. 2) where voltages exceeding 0.5 V result in increased solution uptake in the INENI and voltages less than 0.5 V result in solution being expelled from the INENI. One advantages of the INENI is the ability to accurately control the dosage delivery via applied voltages.

**Figure 3:**
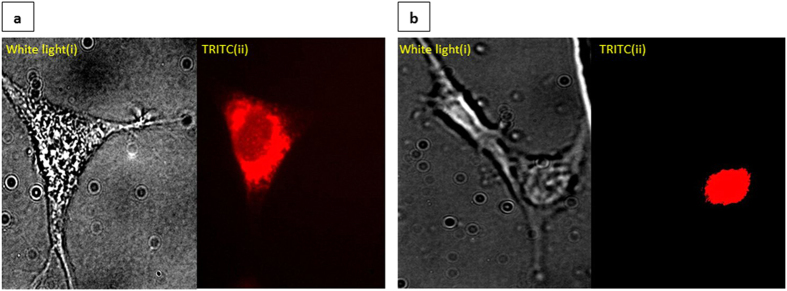
Injection of TRITC into cytoplasm and nucleus (**a**). Upper Left Panel. Injection of tetramethylrhodamine isothiocyanate–dextran into cytoplasm. (i) Left. Optical image of cell. (ii) Right. Fluorescent image of the cell. (**b**) Upper Right Panel. Injection of tetramethylrhodamine isothiocyanate–dextran into nucleus. (i)Left. Optical image of cell. (ii)Right. Fluorescent image of the same cell.

The INENIs were used to inject 50–70fL of tetramethylrhodamine isothiocyanate–dextran into the cytoplasm as well as the nuclei of mouse NIH 3t3 cells. Dextran molecules are hydrophilic polysaccharides that are conjugated to a dye so that the dye does not escape the membrane of nucleus or cell^23^. The INENI was inside the cell for 10 s during injection. After injection, cells were imaged using a fluorescence microscope with Tetramethyl Rhodamine IsoThiocyanate (TRITC) filter (Fig. 3). Our INENI was used to preferentially inject this macromolecule dye inside nucleus or the cytoplasm. By placing the INENI on the targeted region (nucleus or cytoplasm), the technique can be used to elicit spatial localization at the point of injection. The dye solution was collected from a separate container and was injected into each cell. The large molecular weight of dextran allowed dye molecules to stay localized and not escape the cell membrane or the nuclear membrane^13^.

Cell viability experiments were not performed after injection of this dye. The injection of the dye into nucleus or cytoplasm was solely performed to prove that this technique can be used to inject and localize molecules in different compartments of a cell. It is not known whether the dextran conjugated dye at the concentration used had long term toxic effects on cells.

**Figure 4:**
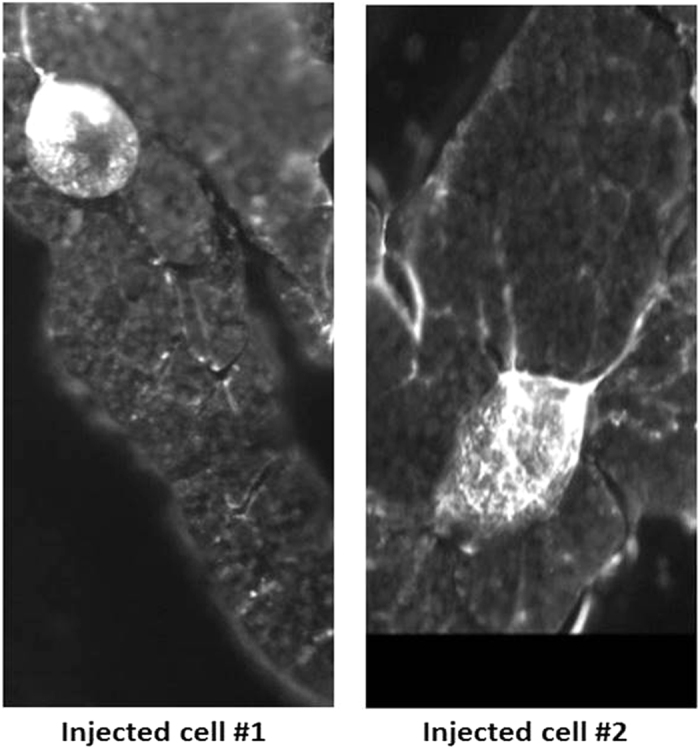
Application of INENI in tissues. Injection of DNA tagged FAM into two representative fat body cells.

Additionally, the technique was used to inject 10 μM DNA tagged Fluorescein amidite (FAM) into several fat body cells, cell number 1 and cell number 2 are shown in Fig. 4. Since DNA is negatively charged, with no injection, DNA tagged FAM did not get into cells.

**Figure 5:**
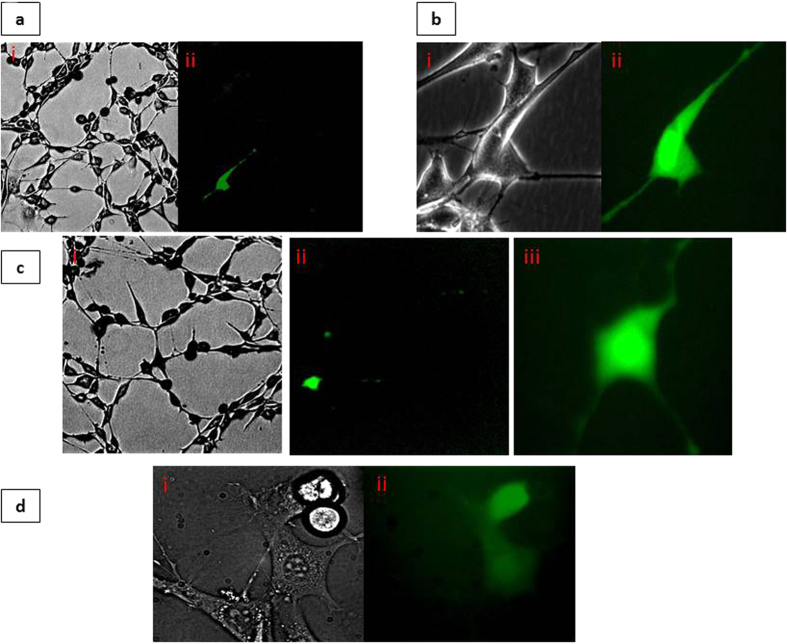
GFP expressing cells 48 hours after plasmid injection using INENIs. (**a**) (i) Bright field image of cell at lower magnification (10x). (ii) Cell fluorescence image using Fluorescein Isothiocyanate (FITC) filter. (**b**) (i) Bright field image of the same cell shown in A under 40x. (ii) Cell fluorescence image using FITC filter under 40x. (**c**) (i) Bright field image of cell at 10x. (ii) The same cell using FITC filter at 10x. (iii) Same cell using FITC filter 40x. (**d**) (i) bright field image of daughter cell expressing GFP 48 hours after transfection. (ii) The same cell using FITC filter.

Figure 5 shows the capability of INENIs in single cell studies, an estimated 1,800 molecules of 3.5 kb plasmid were injected with an INENI inside NIH 3T3 cells and 48 hours after injection cells expressed green fluorescent protein (GFP).

Published works on microinjection indicate that 1,000 molecules of injected plasmid resulted in a faint fluorescent signal, whereas 100,000 molecules resulted in bright fluorescence^24^. Below 1,000 molecules, cells were rarely transfected using microinjection^24^. The same INENI could be used for injection of up to six cells. To have a high throughput within the hour, collection and ejection of plasmid happened inside the chamber where cells resided. Plasmid was combined with DMEM solutions inside a small PDMS chamber and INENIs were not withdrawn from solution each time for pick up. PDMS chambers were designed to minimize the amount of plasmid needed for injection. To maintain cell viability, cells were kept outside of the incubator for no more than one hour. The transfection efficiency of the technique was evaluated to be close to 100%. In a series of two experiments, twelve cells were injected with plasmid, and after 48 hours, 13 cells were fluorescing indicating GFP expression not only in the injected cells, but also in daughter cells (Fig. 5d, Supplementary Figs S1 and S2). Within an hour, up to15 cells could be injected. A negative control was performed where cells residing in the chamber were exposed for one hour to the same concentration of plasmid without any injection. No GFP expression was observed 48 hours after the experiment.

Higher throughput can be achieved by automating the system. By combining the INENI with vision system and registering the INENI and cells with respect to a reference coordinate in the image plane, the movement time of the injector from cell to cell can be reduced to one or two seconds. The transfection time from cell to cell is primarily limited by fluid ejection time (10 seconds) rather than movement of INENI from cell to cell.

## Discussion

In brief, using this technique, we have successfully transfected NIH 3t3 cells with 100% efficiency. Furthermore, fabrication of the nanoinjector is simple and inexpensive. The volume of delivery can be controlled via voltage application. With higher voltages, more liquid enters the INENI and with lower voltages liquid is expelled. This technique can be used to deliver plasmid DNA directly into the nuclei of cells. The INENI requires only the use of a single probe since both electrodes are integrated into the same nanopipette. Hence more space is available, and ergo the INENI offers a simplistic means for direct injection of metered amounts of exogenous material into the confines of a cell cytoplasm and/or nucleus while retaining full cell viability.

The number of plasmid molecules used in this study was 1,800 copies. Since this is well below the 100,000 copies usually used, our technique requires nearly 50 times less plasmid for successful transfection^24^.

We conjecture that the transfection efficiency of this technique reaches 100% due to the small diameter of the nanoinjector compared to microinjectors and direct plasmid insertion into the nucleus rather than to the cytoplasm with little disruption to the nuclear membrane.

The described technique in this study is recognized for potential drug interaction studies at the single cell level as well as single cell analysis.

## Methods

### Fabrication of INENIs

INENIs were fabricated from borosilicate glass capillaries (Sutter Instrument, Novato, CA) using a P-97 puller (Sutter Instrument, Novato, CA).

INENIs were sputter coated with 10 nm layer of iridium followed by 20 nm platinum on one side. The nanoinjectors were oxygen plasma treated at a power of 100 W for 6 min before the experiment. Nanoinjectors were filled with a solution of 1,2-dichloroethane (DCE) containing 10 mM tetrahexyl ammonium bromide. A silver wire coated with AgCl was then inserted into the barrel of the nanoinjector.

### Injection Set up

The injection set-up comprised X, Y, and Z translational micrometer stages for coarse movement (Newport, M-433 for Z movement and M-TSX-1D for X and Y movements) and a nanocube piezo actuator (Physik Instrument, P-280) for finer axial motions of INENI. These stages were placed on an IX71 Olympus microscope via adaptors that were 3D printed in our lab. For current measurement and voltage application a 2116 Keithly Sourcemeter was employed. For pulsing purposes, an Agilent function generator was used (Model number 33220A), and fluorescent image acquisition employed a SBIG camera (Model number ST-7xMEI). The system was operated using custom coded software written in Labview (National Instruments). Volume of liquid entry was measured via the height of solution using ImageJ software and a transmission electron microscopy (TEM) reference grid.

### Cell Culture

NIH3T3 mouse Fibroblasts were purchased from the ATCC and cultured with high glucose DMEM media supplemented with 10% fetal bovine serum (ThermoFisher Scientific). All cells were cultured in 5% CO_2_ at 37 °C and washed with 1x phosphate buffered saline (PBS). Polydimethylsiloxane (PDMS) were made by 10:1 w/w base prepolymer, curing agent formulation. PDMS was cut into small pieces and square holes of 1 mm by 1 mm by 1 mm were created in each PDMS chamber. PDMS chambers were cleaned with 70% ethanol, dried and were placed on top of sterile Petri dishes (Sigma Aldrich). Gridded Petri dish (polymer coated, Ibidi, Germany) was used for tracking cells. Cells at 60–70% confluency were used in experiments.

Wild type Fruit flies were purchased from Bloomington Stock Center and were dissected to isolate fat body cells. Adipocyte tissues were attached to coverslips that were coated overnight with Poly-L-lysine (Sigma-Aldrich). Cells were used immediately after dissection.

### Dye Preparation

Tetramethylrhodamine isothiocyanate–dextran was purchased from Sigma Aldrich. The final concentration of the dye injected was 8–10 mg/mL.

DNA tagged fluorescein was purchased from IDT and was diluted to 10 μM in Schneider’s drosophila medium (Thermofisher scientific). After the injection, DNA tagged FAM was removed and the cells were washed 3 times with PBS and were imaged with the fluorescent camera.

### Plasmid Preparation

pmaxGFP plasmid was purchased from Lonza. Final concentration of plasmid was 0.12 μg/μL. Plasmid was diluted in DMEM and placed in PDMS chamber for further experiments. After the experiment, cells were covered with 10% FBS, 90% DMEM, and 1% antibiotic-antimycotic. (ThermoFisher Scientific).

## Additional Information

**How to cite this article**: Shekaramiz, E. *et al*. Integrated Electrowetting Nanoinjector for Single Cell Transfection. *Sci. Rep.* **6**, 29051; doi: 10.1038/srep29051 (2016).

## Supplementary Material

Supplementary Information

Supplementary Video
