# Supplementary material for: Integrated Electrowetting Nanoinjector for Single Cell Transfection
Source: Sci Rep. 2016 Jul 4;6:29051. doi: 10.1038/srep29051 (PMC4931508; doi:10.1038/srep29051)
Supplement: Supplementary Information [file srep29051-s1.pdf]

Supplementary Information

Integrated Electrowetting Nanoinjector for Single Cell Transfection

Elaheh Shekaramiz[1], Ganeshkumar Varadarajalu[2], Philip J. Day[3], H. Kumar Wickramasinghe[1, 2*]

[1]Department of Biomedical Engineering, University of California Irvine, California, USA
[2]Department of Electrical Engineering, University of California Irvine, California, USA
[3]Manchester Institute of Biotechnology, the University of Manchester, Manchester, UK
https://uk.yahoo.com/

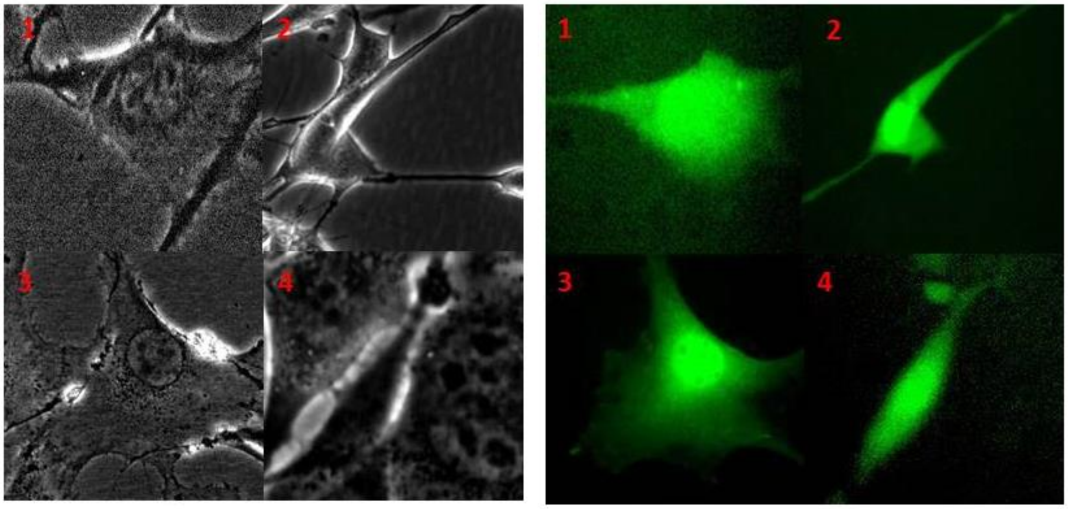

Figure S1. Transfected NIH 3t3 cells. Left panel. Bright field images of transfected NIH 3t3 cells. Right Panel. Fluorescent images of the same transfected cells.

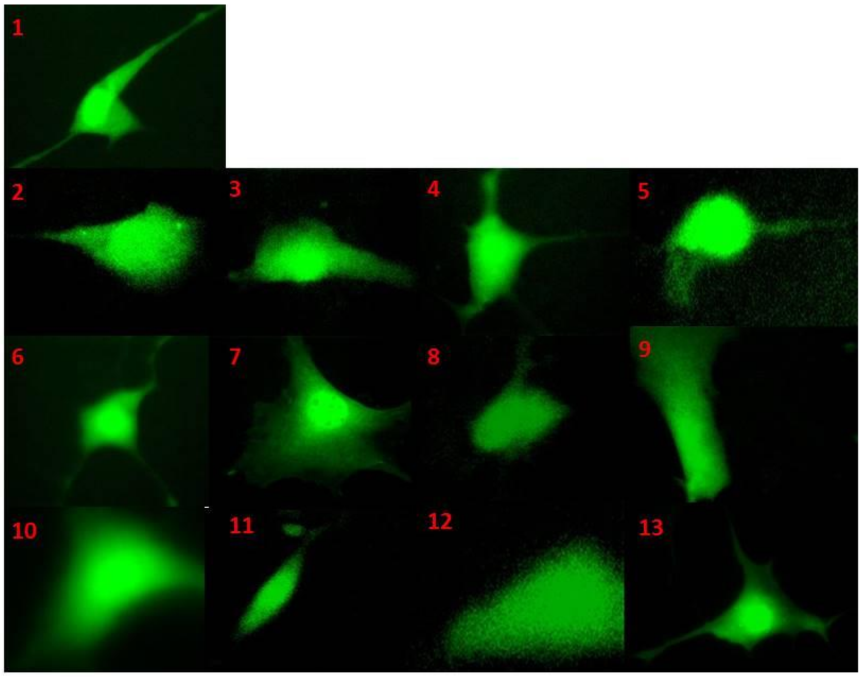

Figure S2. Fluorescent images of one out of two set of transfected cells that expressed green fluorescent proteins.
